# New Drugs for Hepatic Fibrosis

**DOI:** 10.3389/fphar.2022.874408

**Published:** 2022-06-13

**Authors:** Liang Shan, Fengling Wang, Dandan Zhai, Xiangyun Meng, Jianjun Liu, Xiongwen Lv

**Affiliations:** ^1^ Department of Pharmacy, The Second People's Hospital of Hefei, Hefei Hospital Affiliated to Anhui Medical University, Hefei, China; ^2^ Anhui Province Key Laboratory of Major Autoimmune Diseases, Anhui Medical University, Hefei, China; ^3^ Inflammation and Immune Mediated Diseases Laboratory of Anhui Province, Hefei, China; ^4^ The Key Laboratory of Major Autoimmune Diseases, Hefei, China

**Keywords:** anti-hepatic fibrosis drug, hepatic fibrosis, HSCs, inflammation, oxidative stress

## Abstract

The morbidity and mortality of hepatic fibrosis caused by various etiologies are high worldwide, and the trend is increasing annually. At present, there is no effective method to cure hepatic fibrosis except liver transplantation, and its serious complications threaten the health of patients and cause serious medical burdens. Additionally, there is no specific drug for the treatment of hepatic fibrosis, and many drugs with anti-hepatic fibrosis effects are in the research and development stage. Recently, remarkable progress has been made in the research and development of anti-hepatic fibrosis drugs targeting different targets. We searched websites such as PubMed, ScienceDirect, and Home-ClinicalTrials.gov and found approximately 120 drugs with anti-fibrosis properties, some of which are in phase Ⅱ or Ⅲ clinical trials. Additionally, although these drugs are effective against hepatic fibrosis in animal models, most clinical trials have shown poor results, mainly because animal models do not capture the complexity of human hepatic fibrosis. Besides, the effect of natural products on hepatic fibrosis has not been widely recognized at home and abroad. Furthermore, drugs targeting a single anti-hepatic fibrosis target are prone to adverse reactions. Therefore, currently, the treatment of hepatic fibrosis requires a combination of drugs that target multiple targets. Ten new drugs with potential for development against hepatic fibrosis were selected and highlighted in this mini-review, which provides a reference for clinical drug use.

## Introduction

Hepatic fibrosis is one of the most important manifestations of chronic liver injury (Roehlen et al., 2020). At present, the mechanism of its occurrence has not yet been clarified and there is a lack of effective treatment drugs (Gilgenkrantz et al., 2021). Hepatic fibrosis is a necessary process for most chronic liver diseases to develop into cirrhosis. Hepatic fibrosis is a pathological process of abnormal deposition of extracellular matrix (ECM) in liver tissue caused by a persistent injury-repair response, which further leads to abnormal changes in liver structure and function (Boyer-Diaz et al., 2021). The activation of hepatic stellate cells (HSCs) is the central link in the occurrence of hepatic fibrosis, and the inflammatory response to liver cell injury plays a key role in the development of fibrosis (Chen et al., 2021).

**FIGURE 1:**
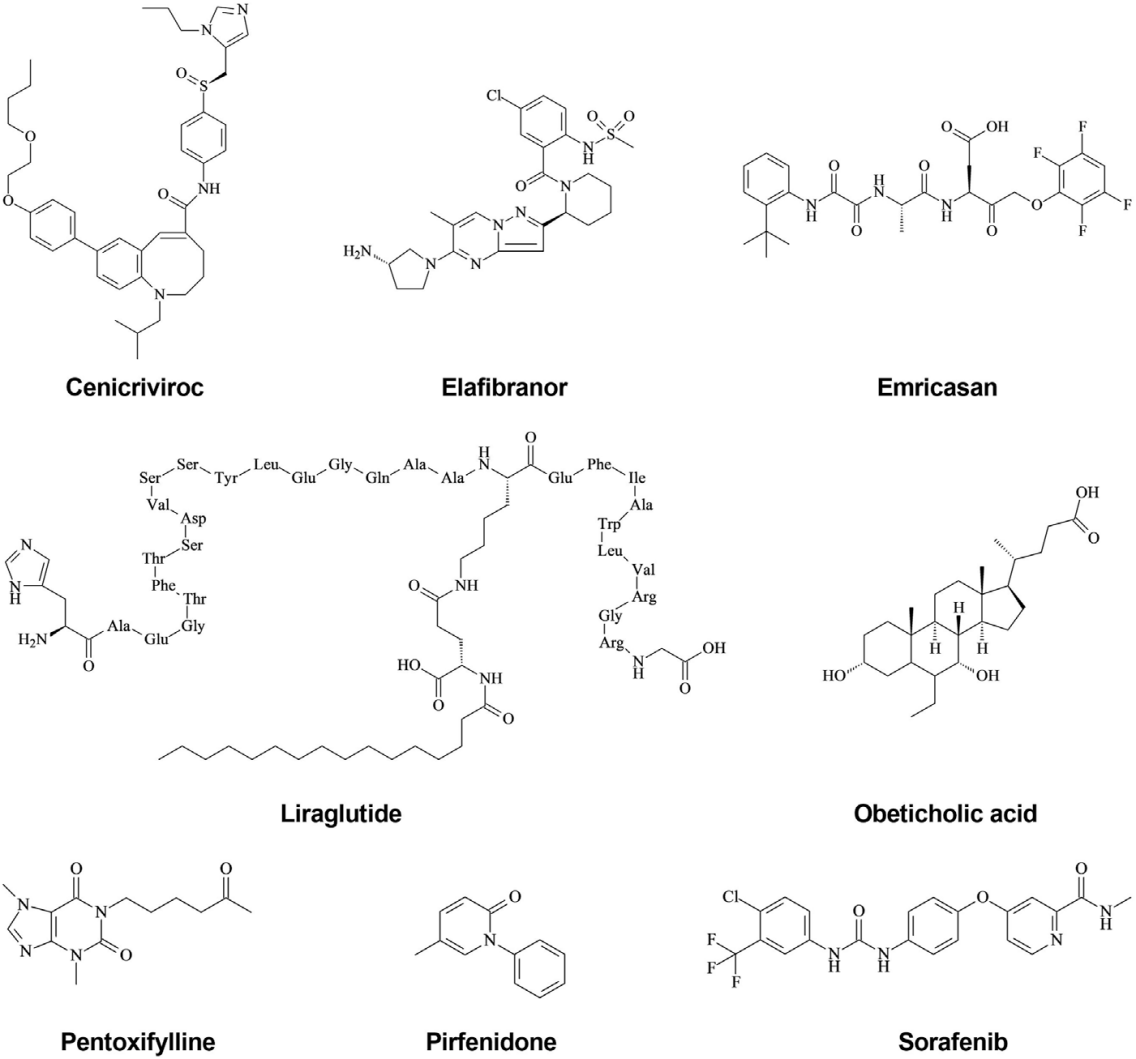
Structural formula of new drugs with anti-hepatic fibrosis effects. (1) Cenicriviroc is a dual antagonist of CCR2/5, which also inhibits both HIV-1 and HIV-2, and displays potent anti-hepatic fibrosis activity. (2) Elafibranor is a double agonist of PPARα/δ, and both animal experiments and clinical trials have found that it has anti-hepatic fibrosis activity. (3) Emricasan is an irreversible pan-caspase inhibitor, which is currently in a phase 2 clinical trial to test its efficacy in treating liver injury and fibrosis, although its clinical trial results are controversial. (4) Liraglutide is a GLP-1 receptor agonist used clinically to treat type 2 diabetes, which functions by ameliorating the progression of NAFLD in patients with T2DM. (5) Obeticholic acid is a potent, selective, and orally active FXR agonist; FXR has been shown to reduce inflammatory mediator expression in HSCs via the induction of PPARγ, suggesting that FXR is an ideal target for the treatment of hepatic fibrosis. (6) Pentoxifylline is an orally active non-selective PDE inhibitor, with anti-inflammatory and anti-proliferation effects. PDE has a strong anti-fibrosis effect but needs more in-depth research. (7) Pirfenidone has broad-spectrum anti-fibrosis effects and is the first drug to demonstrate some efficacy for IPF, which was approved by the FDA in 2008. The mechanism of action of Pirfenidone may be related to the reduction of TGF-β-induced signal transduction pathways. (8) Sorafenib is a multikinase inhibitor of Raf-1, B-Raf, and VEGFR-2, an inhibitor of tyrosine protein kinases that targets the Raf/Mek/Erk pathway. Animal experiments have shown that Sorafenib has anti-fibrosis effects, and the mechanism may be related to inhibiting the TGF-β1/Smad3 pathway.

Hepatic fibrosis is caused by a variety of etiological factors, including alcoholism, viral hepatitis, autoimmune hepatitis, non-alcoholic steatohepatitis (NASH), primary biliary cirrhosis, and primary bile duct cirrhosis (intrinsic and extrinsic factors). Hepatic fibrosis is an inflammation disorder, and cytotoxicity, liver damage, and excessive accumulation of fat can cause the liver inflammatory response. Inflammatory cytokines released by inflammatory cells promote the activation of HSCs, which leads to an increase in ECM release and ultimately causes hepatic fibrosis. Intrinsic factors encompass genetic alterations of cellular pathways leading to the activation of inflammatory pathways such as nuclear factor kappa-light-chain-enhancer of activated B cells (NF-κB), among others. Extrinsic components include inflammatory pathways activated by the liver microenvironment, such as chemokines, cytokines, and adhesion molecules. Although 120 drugs are currently being evaluated for treating hepatic fibrosis, none have yet been approved by the United States Food and Drug Administration (FDA) to treat the disease (Roehlen et al., 2020; Gilgenkrantz et al., 2021). However, many phases Ⅱ and Ⅲ clinical trials are ongoing, and a new chapter for treating hepatic fibrosis is expected in the near future (Table 1) (Figure 1). The main reason for the failure of the 120 drugs is that most are in the stage of animal experiments or clinical trials, with different shortcomings and a lack of adequate data on evidence-based medicine.

**TABLE 1:** List of drugs currently being evaluated in phase Ⅱ and phase Ⅲ clinical trials.

Drug(s)	Mechanism	Research Unit	Research State	Trial Identification
Belapectin	Gal-3	Galectin Therapeutics	Ⅱ	NCT02421094
Cenicriviroc	CCR2/5	Takeda	Ⅲ	NCT03028740
Elafibranor	PPAR-α/δ	Genfit	Ⅲ	NCT02704403
Emricasan	Pan-caspase	Conatus Pharmaceutical	Ⅱb	NCT02686762
Liraglutide	GLP-1	Novo Nordisk	Ⅲ	NCT02654665
Obeticholic acid	FXR	Intercept	Ⅲ	NCT02548351
Pentoxifylline	TNF-α	US Pharm Holdings	Ⅱ	NCT02283710
Pirfenidone	PDE	Marnac	Ⅱ	NCT02161952
Simtuzumab	LOXL2	Gilead	Ⅱ	NCT01707472
Sorafenib	VEGFR-2/PDGF-β	Bayer	Ⅲ	NCT01849588

## Belapectin (Galactoarabino-Rhamnogalacturonate, GR-MD-02)

**FIGURE 2:**
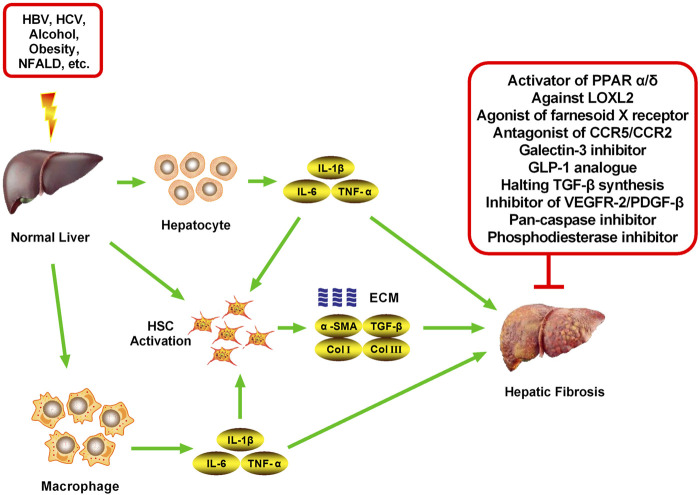
Main targets of new drugs against hepatic fibrosis. Hepatic fibrosis can be induced by various factors, including HBV, HCV, alcohol, obesity, and NFALD, all of which can stimulate the normal liver to induce an inflammatory response. Various cells in the liver, mainly hepatocytes, macrophages, and HSCs, secrete inflammatory cytokines (mainly IL-1β, IL-6, and TNF-α) after receiving stimulation. A large number of inflammatory cytokines continuously stimulate HSCs, inducing activation, proliferation, and the secretion of many fibrosis cytokines, including α-SMA, TGF-β, collagen-I, and Collagen-Ⅲ, which are important components of the ECM. The chronic accumulation of ECM eventually leads to hepatic fibrosis. Inhibiting the activation and proliferation of HSCs and reducing the accumulation of ECM are the most important methods to reverse hepatic fibrosis. Numerous recent studies have found many targets for inhibiting the process of hepatic fibrosis, which has led to the development of novel therapeutic drugs, mainly activators of PPAR α/δ, agonists of FXR, antagonists of CCR2/5, Galectin-3 inhibitor, GLP-1 analog, inhibitors of VEGFR-2/PDGF-β, LOXL2 inhibitor, Pan-caspase inhibitor, a Phosphodiesterase inhibitor, and TGF-β signaling inhibitors.

**FIGURE 3:**
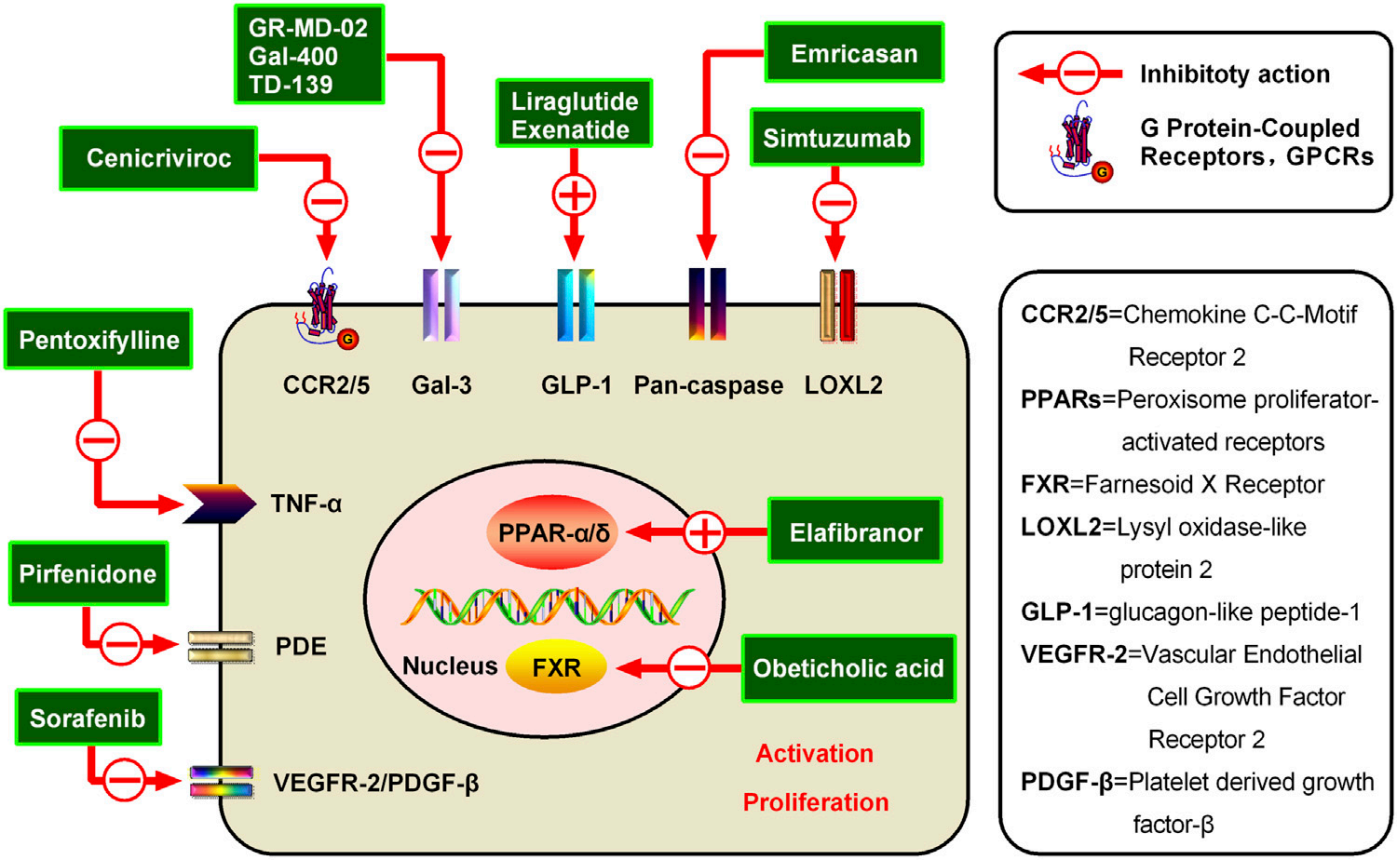
Potential candidates for hepatic fibrosis and their mechanisms of action. Inhibiting the activation and proliferation of HSCs is an important method for the prevention and treatment of hepatic fibrosis. Various representative drugs have emerged for different targets. (1) PPARs are members of the nuclear receptor superfamily, and the PPAR agonist Elafibranor can inhibit liver fibrosis through direct anti-inflammatory effects and indirect improvement of the oxidative stress state. (2) Additionally, a representative FXR receptor agonist, Obeticholic acid, has been shown to reduce liver inflammation and promote ECM degradation to alleviate hepatic fibrosis. (3) Simtuzumab is a monoclonal antibody currently being developed by Gilead for NASH, cirrhosis, and advanced hepatic fibrosis blocking of LOXL2. LOXL2 is a protease that modifies the ECM by promoting the cross-linking of collagen fibers and is believed to play an important role in tumor progression and fibrosis. (4) Emricasan is an irreversible pan-caspase inhibitor, which can reduce the activity of caspases to improve the inflammatory environment and inhibit HSC activation. (5) The GLP-1R agonist Liraglutide can inhibit the formation of ECM and reduce the liver inflammatory response and fibrosis process. (6) Belapectin is a galectin-3 antagonist, which can alleviate hepatic fibrosis and portal hypertension in rats and was found to be safe and well-tolerated in a phase I trial. (7) The CCR2/5 antagonist Cenicriviroc can improve hepatic fibrosis by inhibiting liver inflammation. Cenicriviroc is not only effective for early hepatic fibrosis but also feasible for maintenance treatment in patients with advanced fibrosis. (8) Pentoxifylline is a non-specific PDE inhibitor, which can increase intracellular cAMP concentration and plays an anti-hepatic fibrosis role by inhibiting TNF-α production. (9) Attenuating TGF-β-induced signal transduction pathways can inhibit hepatic fibrosis, such as via Pirfenidone (10) Sorafenib can inhibit VEGFR-2 and PDGF-β to alleviate hepatic fibrosis.

Belapectin is a galectin-3 antagonist developed by Galectin (Rotman and Sanyal, 2017; Neuschwander-Tetri, 2020). Early preclinical studies have shown that Belapectin is a candidate drug for anti-fibrosis research and can reverse the degree of hepatic fibrosis in steatohepatitis mice and prevent collagen deposition before the occurrence of fibrotic cells (Figures 2, 3) (Rotman and Sanyal, 2017; Iacobini et al., 2011). Elevated galactosin-3 levels are associated with NASH and induce hepatic fibrosis in mice (Iacobini et al., 2011; Harrison et al., 2016). Belapectin, an inhibitor of galactosin-3, can alleviate hepatic fibrosis and portal hypertension in rats and was shown to be safe and well-tolerated in a phase I trial (NCT01899859) (Harrison et al., 2016). Galectin is currently in a phase III trial to evaluate Belapectin for the prevention and treatment of nonalcoholic fatty liver disease (NAFLD), portal hypertension, fibrosis, psoriasis, liver function decline, and other diseases (Chalasani et al., 2020).

Belapectin single and three weekly repeated at 2, 4, and 8 mg/kg demonstrated no meaningful clinical differences in treatment-emergent adverse events, vital signs, electrocardiographic findings, or laboratory tests. Pharmacokinetic parameters showed a dose-dependent relationship, with evidence of drug accumulation following 8 mg/kg (Harrison et al., 2016). Results of a 52-weeks phase Ⅱb trial suggest that Belapectin is an effective anti-fibrotic drug for compensatory NASH cirrhosis (NCT02462967) (Chalasani et al., 2020). Because the treatment can take many years, there is an urgent need for effective drug candidates with good safety and tolerability (NCT02421094) (Harrison et al., 2018b). Overall, the clinical success of Belapectin indicates a promising path for the continued clinical development of Belapectin in compensatory NASH cirrhosis, which could make it the first anti-fibrosis drug candidate to win approval from NASH regulators (Chalasani et al., 2018).

## Cenicriviroc (TAK-652, TBR-652)

Cenicriviroc is a chemokine receptor 2/5 (CCR2/5) antagonist that acts differently from previous anti-HIV1 drugs and holds great promise in the field of anti-AIDS drugs (Figure 1) (Friedman et al., 2016; Friedman et al., 2018). The formula of Cenicriviroc is C_41_H_52_N_4_O_4_S, with a molecular weight of 696.94. Studies have found that monocyte chemokine protein-1 (MCP-1), chemokine C-C motif chemokine 2 (CCL2), and regulated upon activation, normal T cells expressed and secreted (RAN-TES) can promote the aggregation of monocytes/macrophages in blood to liver inflammatory sites through CCR2/5, as well as produce various cytokines, such as transforming growth factor-β (TGF-β), tumor necrosis factor-α (TNF-α), interleukin-1β (IL-1β), and IL-6, to further activate HSCs and generate ECM, which leads to the formation of hepatic fibrosis (NCT02217475) (Friedman et al., 2016; Lefebvre et al., 2016a; Lefebvre et al., 2016b). Additionally, CCR2 and CCR5 are highly expressed in activated HSCs, which directly or indirectly mediate various biological functions of HSCs after binding with its ligand and participate in the formation of hepatic fibrosis (NCT02217475) (Lefebvre et al., 2016a; Lefebvre et al., 2016b). Therefore, CCR2 and CCR5 have become important targets for anti-fibrosis therapy (Figures 2, 3).

Cenicriviroc is a novel oral drug developed in collaboration between Takeda and Tobira Therapeutics. The plasma half-life of Cenicriviroc is 30–40 h, and it can be administered once a day (NCT02217475) (Lefebvre et al., 2016a; Friedman et al., 2016). Cenicriviroc has shown good safety, providing new ideas and methods for the clinical prevention and treatment of hepatic fibrosis (Lefebvre et al., 2016a; Friedman et al., 2016). Animal experiments have shown that Cenicriviroc significantly alleviates thioacetamide (TAA)-induced hepatic fibrosis in rats, mainly by inhibiting HSC synthesis of collagen Ⅰ, thereby inhibiting collagen deposition in the liver and reducing liver tissue inflammation (Lefebvre et al., 2016b; Kruger et al., 2018). Therefore, Cenicriviroc may represent an effective drug against hepatic fibrosis.

Clinical studies have found that Cenicriviroc has excellent pharmacokinetic properties in the human body and is well tolerated without causing dose-limiting adverse reactions (Friedman et al., 2016; Friedman et al., 2018). Cenicriviroc is also well absorbed and slowly eliminated (Anstee et al., 2020; Reimer et al., 2020). Additionally, the majority of adverse reactions were mild (grade 1 or 2) and dose-independent, with gastrointestinal disturbances (63%) and systemic adverse reactions (37%) being the most common. Grade 3 adverse events (abscesses) were reported by one subject in each of the placebo and 75 mg (qd) groups, but they were not considered to be related to the product. There were no grade 4 adverse reactions, severe adverse reactions, death, or withdrawal from the study due to adverse reactions (Sumida and Yoneda, 2018; Sumida et al., 2019). A phase II trial showed that Cenicriviroc rapidly blocks CCR2 and CCR5 (Sumida and Yoneda, 2018; Sumida et al., 2019), and it has been shown that 150 mg Cenicriviroc can be used to treat mild and moderate liver injury, with good tolerance. The pharmacokinetic data of Cenicriviroc is relatively complete and it represents a promising drug for treating hepatic fibrosis (Lefebvre et al., 2016a). Cenicriviroc is currently in a phase III trial (NCT03028740) (Sumida and Yoneda, 2018; Pedrosa et al., 2020).

## Elafibranor (GFT505)

Elafibranor is a double agonist of peroxisome proliferator-activated receptors α/δ (PPARα/δ) developed by Genfit, France; its molecular formula is C_22_H_24_O_4_S, and its molecular weight is 384.489 (Boeckmans et al., 2019; Cheng et al., 2019). The chemical structure is shown in Figure 1. Elafibranor alleviates NASH symptoms through various mechanisms, including increased fatty acid oxidation, improved lipid profile, increased insulin sensitivity, and anti-inflammatory and anti-fibrosis effects (Figures 2, 3) (Alukal and Thuluvath, 2019; Ratziu et al., 2019).

Animal experiments have shown that Elafibranor administration can effectively reduce hepatic steatosis, inflammation, fibrosis, and the level of liver dysfunction biomarkers, as well as inhibit the expression of pro-inflammatory and pro-fibrosis genes; it is effective in both the prevention and treatment of hepatic fibrosis (Schuppan et al., 2018; Baandrup Kristiansen et al., 2019; Roth et al., 2019). Elafibranor treatment protects against hepatic steatosis and inflammatory progression (Ratziu et al., 2016; Baandrup Kristiansen et al., 2019), and has shown preventive and therapeutic effects on CCl_4_-induced hepatic fibrosis in rats (Schuppan et al., 2018). In pharmacokinetic studies conducted in rats, Elafibranor and its metabolites were rapidly excreted into bile and underwent extensive enterohepatic circulation (Schuppan et al., 2018). High concentrations of Elafibranor and/or its metabolites were detected in the liver and intestines, with little distribution in other tissues (Schuppan et al., 2018; Roth et al., 2019).

No toxicity or carcinogenesis of Elafibranor was found in long-term animal toxicity tests (Roth et al., 2019; Gore et al., 2020). From 2011 to 2015, Genfit completed a series of phase I clinical trials of Elafibranor, which were found to be both safe and well-tolerated. The safety of Elafibranor is generally good in many clinical trials, and no serious adverse events have occurred to date (Boeckmans et al., 2019; Cheng et al., 2019; Alukal et al., 2016). In completed phase II clinical trials, Elafibranor has shown a certain therapeutic efficacy and good safety and tolerability (Ratziu et al., 2016; Alukal and Thuluvath, 2019). Fast-track approval was granted by the FDA in February 2014, and a phase III clinical trial began in March 2016 (Ratziu et al., 2016; Alukal and Thuluvath, 2019). In January 2018, Genfit announced that the FDA had approved Elafibranor for the pediatric study program and would begin a clinical trial for the treatment of pediatric NASH. Elafibranor is currently in phase III of the clinical trial, and subgroup analyses of results support its potential efficacy in patients with severe NASH (Boeckmans et al., 2019; Cheng et al., 2019). Currently, there is no approved effective drug for NASH (Alukal and Thuluvath, 2019; Boeckmans et al., 2019). Of nearly 200 candidates in development worldwide, Elafibranor is one of the most highly anticipated drugs for NASH to hit the market.

In a 52-weeks phase Ⅱ study in patients with NASH without cirrhosis, 276 patients were randomly assigned to receive Elafibranor at either 80 mg or 120 mg daily or a placebo. Even though the trial was not designed with antifibrotic goals, it is worth noting that among the patients who responded to 120 mg Elafibranor (n = 17), there was a reduction in fibrosis (*p* < 0.001) compared to non-responders to the same regimen. However, at present, there are limited pharmacokinetic data on this drug in the human body, which needs further study (Ratziu et al., 2016). On 11 May 2020, Genfit announced that in its phase III trial of Elafibranor in the treatment of NASH, the drug failed to significantly improve patients’ NASH histological symptoms, and in some cases, exacerbated hepatic fibrosis compared to placebo (Guaraldi et al., 2020; Shen and Lu, 2021). A total of 1070 patients with NASH were randomized (2:1) to receive Elafibranor 120 mg/day or a placebo (Guaraldi et al., 2020; Shen and Lu, 2021). After 72 weeks, 19.2% and 14.7% of patients treated with Elafibranor and placebo had improved NASH histology without worsening hepatic fibrosis, respectively, 24.5% and 22.4% of patients with at least a grade of fibrosis improvement, however, these results were not statistically significant. The safety of Elafibranor was consistent with the results of previous studies (Guaraldi et al., 2020; Shen and Lu, 2021) in that no adverse effects, such as increased fluid retention and heart failure, were mediated by PPARγ (Guaraldi et al., 2020; Shen and Lu, 2021).

## Emricasan (IDN-6556, PF-03491390)

Emricasan is a pioneering and irreversible pan-caspase inhibitor that is orally administered (Figure 1) (Garcia-Tsao et al., 2019; Barreyro et al., 2015). The formula of Emricasan is C_26_H_27_F_4_N_3_O_7_, and its molecular weight is 569.50. Emricasan can be retained in the liver for a long time and can reduce the activity of caspases, which mediate inflammation, cell death, and apoptosis (Garcia-Tsao et al., 2019; Barreyro et al., 2015; Gracia-Sancho et al., 2019). By reducing the activity of these enzymes, Emricasan may block the development of liver disease (Gracia-Sancho et al., 2019; Shiffman et al., 2019). Emricasan, developed by Conatus, has been shown in preclinical studies to reduce apoptosis, improve the inflammatory environment and inhibit HSC activation (Figures 2, 3) (Barreyro et al., 2015; Gracia-Sancho et al., 2019; Shiffman et al., 2019).

Emricasan reduced liver damage in NASH but had no significant effect on metabolic disorders (Frenette et al., 2019; Harrison et al., 2020). Emricasan was previously demonstrated to inhibit some of the liver enzymes which lead to liver inflammation and fibrosis (Frenette et al., 2019; Harrison et al., 2020). In a mouse model of NASH, treatment with Emricasan attenuated hepatic fibrosis and the activation of HSCs (Barreyro et al., 2015; Gracia-Sancho et al., 2019). Emricasan is currently in a phase Ⅱ clinical trial to test its efficacy in treating liver injury and fibrosis through chronic HCV infection (Frenette et al., 2019). In two previous trials, Emricasan was both well-tolerated and safe, and the results were consistent with those of 19 previous clinical studies (Frenette et al., 2019; Shiffman et al., 2019). Emricasan treatment was safe and well-tolerated, with adverse events, severe events, abnormal experimental results, vital signs, cancers, and infections occurring at similarly low rates in the Emricasan treatment group and placebo groups (Frenette et al., 2019; Shiffman et al., 2019; Harrison et al., 2020). The most common adverse reactions in the Emricasan group were headache (16%), nausea (14%), and fatigue (9%) (Frenette et al., 2019; Shiffman et al., 2019; Harrison et al., 2020). Results from a multicenter phase b clinical trial suggest that Emricasan improves liver function in patients with severe cirrhosis (NCT02686762) (Garcia-Tsao et al., 2020). Moreover, a 28-days randomized clinical trial of Emricasan assessed the efficacy, safety, and tolerability of Emricasan in subjects with NAFLD, in which the subjects were randomized to Emricasan 25 mg twice daily or a matching placebo. The results showed that Emricasan decreased ALT and biomarkers in subjects with NAFLD and raised AST after 28 days (NCT02077374). Emricassan has a high first-pass metabolism, but the current pharmacokinetic data are mainly from animal experiments, with a lack of complete human experimental data (Garcia-Tsao et al., 2019).

## Liraglutide (Victoza)

Liraglutide is a Glucagon-Like Peptide (GLP-1) analog developed by Novo Nordisk to decrease blood sugar in patients with type 2 diabetes mellitus (T2DM) (Figure 1) (Kahal et al., 2014; Zoubek et al., 2017). The formula of Liraglutide is C_172_H_265_N_43_O_51_, and its molecular weight is 3751.26. GLP-1 can increase insulin release, decrease glucagon secretion, reduce hepatic steatosis, and improve hepatic fibrosis (Fagone et al., 2016; Briand et al., 2020). GLP-1R agonists can reduce liver cell apoptosis and endoplasmic reticulum stress through various mechanisms. Previuous studies of mechanisms have been proposed, including an increase in cyclic adenosine monophosphate (cAMP) production, activation of an AMP-activated protein kinase (AMPK)-dependent pathway in hepatocytes increasing fatty acid oxidation and decrease in lipogenesis, and/or an increase in hepatic insulin signaling and sensitivity with GLP-1 and subsequent improvement of the hepatic glucose metabolism. Additionally, Liraglutide has been found to reduce fatty acid accumulation, in mice fed a high-fat diet by enhancing autophagy and reducing endoplasmic reticulum stress-related apoptosis. Furthermore, the GLP-1R agonist can reduce liver cell apoptosis and endoplasmic reticulum stress by inhibiting activation of the NACHT, LRR and PYD domains-containing protein 3 (NALP3) inflammasome and NF-κB signaling pathway. Besides, Liraglutide can promote liver glucose and lipid metabolism, and inhibit the secretion of inflammatory cytokines, which may explain its ability to alleviate the process of hepatic fibrosis (Kahal et al., 2014; Zoubek et al., 2017; Fagone et al., 2016). Studies have also shown that Liraglutide can inhibit the formation of ECM, reduce the liver inflammatory response and fibrosis process, and slow and improve the progression of NAFLD in patients with T2DM (Figures 2, 3) (Kahal et al., 2014; Fagone et al., 2016; Zoubek et al., 2017).

The results of animal experiments showed that Liraglutide could significantly reduce collagen fibers in NAFLD models (Fagone et al., 2016; Choi et al., 2019; Briand et al., 2020). Moreover, Liraglutide significantly reduced levels of inflammatory factors, such as IL-6 and TNF-α, and liver fibrosis factors in the NAFLD model group (Fagone et al., 2016; Choi et al., 2019; Briand et al., 2020). Liraglutide may play an important role in NAFLD by activating the SIRT1/AMPK pathway, regulating key regulatory molecules of lipid synthesis and metabolism, and inhibiting the *de novo* synthesis of fatty acids (Kahal et al., 2014). Early intervention with Liraglutide can reduce blood glucose, inhibit fat synthesis, reduce insulin resistance, inflammation, fibrosis, and oxidative stress damage, thereby alleviating NAFLD, which may be related to the activation of SIRT1/AMPK and its downstream genes (Kahal et al., 2014). These results indicate that Liraglutide may represent a potential drug for the treatment of NAFLD, with SIRT1 as a potential therapeutic target.

Recently, in addition to its good hypoglycemic effect, Liraglutide has also been shown to inhibit myocardial fibrosis, renal fibrosis, and hepatic fibrosis (Kahal et al., 2014). Current studies suggest that inflammation is the core cause of hepatic fibrosis, which is mainly associated with NF-κB activation, and initiation of the NF-κB signaling pathway (Gilgenkrantz et al., 2021). Therefore, inhibiting NF-κB mitigates various inflammatory liver diseases, including hepatic fibrosis. Liraglutide may play anti-inflammatory and anti-fibrosis roles by inhibiting the activation of NF-κB and reducing the production of superoxide (Fagone et al., 2016). Liraglutide can also reduce the degree of tissue collagen deposition and improve tissue fibrosis (Armstrong and Newsome, 2015; Choi et al., 2019; Briand et al., 2020), and may play an anti-fibrosis role by regulating some important links in the fibrotic process (Armstrong et al., 2015; Armstrong et al., 2016; Gaborit et al., 2016; Choi et al., 2019). The data from genetic toxicity studies show that Liraglutide is not toxic in the human body (Armstrong et al., 2015; Armstrong et al., 2016; Gaborit et al., 2016). Results of a phase II multicentered trial showed improvement in NASH and no further increase in hepatic fibrosis in 39% of participants in the treatment group compared to only 9% in the placebo group (NCT02654665). Pharmacokinetic studies have shown that about 99% of Liraglutide binds to albumin in the body, allowing it to escape glomerular filtration and extend its duration of action (Alruwaili et al., 2021). It is expected to be one of the candidate drugs for the treatment of hepatic fibrosis in the future.

## Obeticholic Acid (INT-747, 6-ECDCA, 6α-Ethylchenodeoxycholic Acid)

Farnesoid X Receptor (FXR) is a member of the nuclear receptor superfamily and is classified as NR1H4 (Figure 1) (Eslam et al., 2019; Anfuso et al., 2020). FXR is mainly expressed in the enterohepatic system, FXRα1/2 and FXRα3/4 are expressed in the liver, while FXRα3/4 is mainly expressed in the intestine. FXR is widely involved in the pathophysiological processes of many diseases in the enterohepatic system (Armstrong et al., 2015; Gawrieh et al., 2019). Activated FXR plays a protective role in various chronic liver diseases (Brunt et al., 2019; Eaton et al., 2020), and FXR agonists include Obecholic Acid, GW4064, WAY-362450, PR20606, GS9674, and LJN452, which are a batch of synthetic or semi-synthetic FXR agonists. Among them, GW4064 and Obecholic Acid are widely used. The formula of Obecholic Acid is C_26_H_44_O_4_, and its molecular weight is 420.63. GW4064 is a synthetic nonsteroidal FXR agonist (Figures 2, 3).

FXR has been found to be associated with hepatic fibrosis, cirrhosis, and portal hypertension (Younossi et al., 2019; Hindson, 2020; Siddiqui et al., 2020). The activation of HSCs is a key factor in the development of hepatic fibrosis, and it has been previously reported that FXR activation mitigates hepatic inflammation. One mechanism by which FXR reduces inflammatory mediator expression in HSCs is via the induction of PPARγ. Additionally, FXR activation represses gluconeogenesis, TG synthesis, and VLDL export via SHP, a primary FXR-responsive gene, which contributes to regulating FXR target genes. FXR modulates many genes by regulating SHP. Besides, regulation of the MMP/TIMP balance is essential for the transition of the physiological ECM into pathological ECM. The FXR agonist Obecholic Acid has been shown to induce MMP2–9 activity and a dose-dependent reduction of collagen. Therefore, FXR inhibits HSC activation by activating the PPARγ and SHP-TIMP pathways. Additionally, FXR improves portal hypertension by increasing eNOs activity and reducing vascular remodeling (Anfuso et al., 2020). These findings, together with the regulation of metabolic and inflammatory functions, strongly suggest that FXR is an ideal target for the treatment of hepatic fibrosis, cirrhosis, and portal hypertension (Brunt et al., 2019; Gawrieh et al., 2019; Younossi et al., 2019). Studies have shown that the FXR agonist Obecholic Acid can reduce liver inflammation and fibrosis in TAA-induced toxic cirrhosis, and even reverse hepatic fibrosis (Anfuso et al., 2020). FXR inhibits the negative regulator NF-κB by up-regulating IκBα, resulting in reduced expression of pro-fibrotic cytokines and related markers of hepatocyte transformation, thereby weakening the effect of inflammatory cytokines (Younossi et al., 2019).

Hepatic fibrosis is caused by persistent liver injury and is characterized by inflammation, activation of HSCs, accumulation of ECM, and destruction of the liver structure. HSCs proliferate and transform into myofibroblasts, which deposit collagen I and fibronectin in the ECM and eventually produce fibrogenic cytokines (Brunt et al., 2019; Eslam et al., 2019; Anfuso et al., 2020). FXR prevents the activation of HSCs through FXR-PPARγ or FXR-SHP-TIMP pathways. In animal models of hepatic fibrosis induced by TAA, bile duct ligation (BDL), and CCl_4_, Obecholic Acid significantly reduced the expression of hepatic fibrosis genes and proteins, as well as the area of hepatic fibrosis parenchymal tissue (Eslam et al., 2019; Anfuso et al., 2020). The FXR agonist Obecholic Acid has also been shown to increase the interaction between FXR and Smad3 and alleviate CCl_4_-induced liver injury and fibrosis. Studies have shown that FXR gene knockout mice can increase liver inflammation and fibrosis, suggesting that the loss of FXR function is more likely to induce liver inflammation and fibrosis. Clinical trials also showed that Obecholic Acid significantly improves hepatic fibrosis, ballooning degeneration, steatosis, and lobular infiltration (Brunt et al., 2019; Younossi et al., 2019). In addition to Obecholic Acid, other FXR agonists such as PX-102, Way-362450, and EDP-305 have been shown to have protective effects against diet-induced fibrosis such as MCD (Anfuso et al., 2020). Taken together, these preclinical studies support the anti-fibrosis effects of FXR agonists.

The FXR agonist obecholangitis was approved by the FDA on 27 May 2016, for treating primary biliary cholangitis (PBC) in adults (Eaton et al., 2020). Results from two phase Ⅱ clinical trials showed that Obecholic Acid significantly improved the disease activity scores in patients with NAFLD, as well as steatosis, lobular inflammation, ballooning degeneration, and hepatic fibrosis (Younossi et al., 2019; Anfuso et al., 2020). The results of a phase II clinical trial (NCT00501592) showed that Obeticholic Acid significantly improved insulin sensitivity in patients with T2DM complicated with NAFLD, significantly improved hepatic fibrosis, and liver enzymology indexes (*p* < 0.05). Additionally, there were no adverse reactions during the trial, and both safety and tolerability were good. However, elevated serum alkaline phosphatase and total cholesterol concentrations in patients during the study should be seriously considered.

Recently, a phase III clinical trial of Obecholic Acid in the treatment of NASH with fibrosis has been completed (Younossi et al., 2019; Hindson, 2020). In the trial, 18% of patients who received 10 mg Obecholic Acid and 23% who received 25 mg Obecholic Acid showed improvement in hepatic fibrosis. Another double-blind placebo-controlled trial investigating the efficacy and safety of Obecholic Acid in the treatment of type 2 diabetes complicated by NAFLD also demonstrated a significant reduction in hepatic fibrosis markers in patients taking 25 mg Obecholic Acid (Younossi et al., 2019; Hindson, 2020). These clinical studies demonstrate that the FXR agonist Obecholic Acid is promising for the treatment of hepatic fibrosis. Recently, some scholars have suggested that specific micro RNA is also a target gene of FXR, which can regulate the process of hepatic fibrosis. After mice and human HSCs were treated with the FXR agonist GW4064, miR-29a levels increased, ECM accumulation decreased, and hepatic fibrosis was alleviated. Additionally, the level of FXR in liver tissue of patients with severe hepatic fibrosis was decreased and the level of miR-199a-3p was increased, while the expression of miR-199a-3p was inhibited after activation of FXR, which further inhibited the proliferation of HSCs and alleviated hepatic fibrosis.

Since Obecholic Acid was marketed in May 2016, 19 deaths have been confirmed, of which 8 have provided the cause of death information and seven patients with moderate to severe liver dysfunction may have been caused by the use of the drug beyond the recommended dose (Younossi et al., 2019). The FDA recommends that physicians determine a patient’s baseline liver function before administering Obecholic Acid and strictly adhere to the approved dosing regimen (Younossi et al., 2019; Siddiqui et al., 2020). The most common adverse reactions shown in clinical trials were severe pruritus, which resulted in discontinued treatment in some patients at higher doses. Some patients also experienced fatigue, abdominal pain and discomfort, arthralgia, constipation, elevated blood sugar, elevated blood lipids, dizziness, and dysarthria, which may be caused by cerebral ischemia. The observed increase in LDL and decrease in HDL suggest that this drug may lead to the occurrence of cardiovascular and cerebrovascular events (Eslam et al., 2019; Younossi et al., 2019; Siddiqui et al., 2020). However, these adverse reactions can be effectively alleviated after dose control and medication regimen adjustment. Obecholic Acid is subject to enterohepatic circulation, and the pharmacokinetic parameters of the active metabolites show that food may increase its absorption (Wang et al., 2021).

## Pentoxifylline

Pentoxifylline, a derivative of Methylxanthine, is a non-specific phosphodiesterase inhibitor developed by Pharm Holdings, with various pharmacological characteristics, mainly used in cerebrovascular diseases (Figure 1) (Oberti et al., 1997; Desmoulière et al., 1999; Chooklin and Perejaslov, 2003). The formula of Methylxanthine is C_13_H_18_N_4_O_3_, and its molecular weight is 278.31. Recent studies have found that Pentoxifylline also has a strong anti-fibrosis effect, which can effectively inhibit hepatic fibrosis, kidney fibrosis, and skin scar formation (Peterson, 1993; Pinzani et al., 1996; Verma-Gandhu et al., 2007). Pentoxobromine is a first-line drug for the treatment of AH, which has anti-inflammatory, anti-hepatic fibrosis, and immunological regulation effects (Louvet et al., 2008; Ali et al., 2018). However, the specific mechanism of Pentoxifylline is still unclear (Figures 2, 3).

The Hedgehog signaling pathway plays an important role in cell differentiation and proliferation during embryonic development. Recent studies have found that the hedgehog signaling pathway is involved in the repair of liver injury and the occurrence of hepatic fibrosis (Solhi et al., 2022). Furthermore, Pentoxifylline may block the activation of HSCs by inhibiting the hedgehog signaling pathway and inhibit the occurrence of hepatic fibrosis in Schistosoma, and is therefore expected to be an effective drug for the prevention and treatment of Schistosoma in clinical practice. Additionally, PPAR-α and NF-κB P65 have been studied in many fields and are closely related to AH due to their anti-inflammatory, anti-oxidation, and anti-hepatic fibrosis effects, and regulation of fat metabolism (Ali et al., 2018). Current studies have found that Pentoxifylline can treat AH in rats, and its mechanism may be related to the upregulation of PPAR-α expression and downregulation of NF-κB P65 expression (Satapathy et al., 2007; Ali et al., 2018).

Through non-selective inhibition of phosphodiesterase, Pentoxifylline reduces intracellular cAMP hydrolysis to 5-AMP and increases intracellular cAMP concentrations, inducing corresponding changes in cells and inhibition of the calcium ion influx (Pinzani et al., 1996). Pentoxifylline improves hemorheology in many complementary ways, including reducing blood and plasma viscosity, reducing plasma fibrinogen, and promoting fibrinolysis. Additionally, Pentoxifylline improves blood permeability in tissues by enhancing the plasticity of erythrocytes and reducing neutrophil activation. Pentoxifylline has a protective effect on the liver by improving liver hemorheology and has anti-inflammatory and anti-fibrosis effects (Zein et al., 2012; Park et al., 2014). As an anti-inflammatory and anti-fibrosis drug, Pentoxifylline can inhibit the production of TNF-α, a pro-inflammatory cytokine, at 400 mg/day, 3 times per day for 28 days, which can effectively delay the disease progression in patients with severe alcoholic liver disease. Additionally, the anti-fibrosis and anti-inflammatory effects of Pentoxifylline have certain protective effects on the liver. The mechanism of action of Pentoxifylline is completely different from that of other commonly used liver protective drugs, so a combination of Pentoxifylline can produce complementary effects (Lebrec et al., 2010; Zein et al., 2012; Park et al., 2014).

As a non-specific phosphodiesterase inhibitor, Pentoxifylline has anti-inflammatory and anti-fibrosis effects, improves hepatic hemorheology, inhibits hyperplasia, and has certain effects on the prevention and treatment of cirrhosis and reduces mortality of liver disease (Ali et al., 2018). Existing clinical evidence shows that Pentoxifylline combined with corticosteroids can reduce the risk of death in severe alcoholic hepatitis (Louvet et al., 2008). Pentoxifylline can improve the histological characteristics of NAFLD/NASH, prevent hepatic steatosis, and improve liver function in patients with non-dyslipidemia NAFLD (Satapathy et al., 2007; Zein et al., 2012). Furthermore, Pentoxifylline can improve the survival rate of patients with HRS and is expected to be a therapeutic drug for HRS. Pentoxifylline hit the market earlier, and its efficacy, toxicity, and pharmacokinetic data are relatively complete. Previous studies have found that Pentoxifylline can be rapidly and widely absorbed from the gastrointestinal tract of animals and humans and rapidly metabolized systemically (Ward and Clissold, 1987).

## Pirfenidone

Pirfenidone, chemically known as 5-Methyl-N-phenyl-2-1H-pyridone, is a new kind of pyridinone compound with a broad spectrum of anti-fibrosis effects, which can prevent and reverse the formation of fibrosis and scars (Figure 1) (Grizzi, 2009; Di Sario et al., 2002). The formula of Pirfenidone is C_12_H_11_NO, and its molecular weight is 185.22. Pirfenidone, marketed by Shionogi in 2008 and approved by the FDA, is the first drug to demonstrate some efficacy for idiopathic pulmonary fibrosis (IPF) in a repeated, randomized, placebo-controlled phase III clinical trial (Armendáriz-Borunda et al., 2006; Verma et al., 2017; Sandoval-Rodriguez et al., 2020). Moreover, it also has a good effect on fibrosis diseases, such as renal interstitial fibrosis and hepatic fibrosis. However, its mechanism of action for treating IPF is still unclear (Benesic et al., 2019; Zhang et al., 2019). Current studies have shown that Pirfenidone can reduce the proliferation of lung fibroblasts and their differentiation into myofibroblasts by attenuating TGF-β-induced signal transduction pathways (Smad3, P38, and Akt), decreasing the expression of recombinant Heat Shock Protein 47 (HSP47) induced by TGF-β, reducing the expression of α-SMA and collagen I (Figures 2, 3) (Di Sario et al., 2002; Benesic et al., 2019; Sandoval-Rodriguez et al., 2020).

Pirfenidone can inhibit the proliferation of hepatocytes and promote hepatocyte apoptosis by inhibiting the Wnt/β-Catenin signaling pathway, which is an effective oral small-molecule drug for the treatment of fibrosis. Many studies have found that Pirfenidone has important anti-inflammatory and anti-fibrosis effects *in vivo* and *in vitro* (Di Sario et al., 2002; Grizzi, 2009; Benesic et al., 2019). Pirfenidone can regulate TGF-β and TNF-α and inhibit fibroblast proliferation and collagen synthesis (García et al., 2002; Salah et al., 2019; Ullah et al., 2019). Moreover, Pirfenidone can significantly alleviate hepatic fibrosis induced by CCl_4_ in mice; this effect is closely related to the mechanism of reducing expression levels of PI3K and PKB in the mouse liver (Grizzi, 2009; Benesic et al., 2019). These results suggest that Pirfenidone is a potential therapeutic agent for hepatic fibrosis.

Of the 978 adverse events reported, 17% were nausea and vomiting, 16% were diarrhea, 10% were fatigue, 9% were loss of appetite, 6% were dyspepsia, 6% were rash (n = 59), 5% were headache, and 10% were others (Maher et al., 2020). The incidence of other adverse events was lower than 5%; the most common was acute respiratory failure (2 cases), indicating that the disease is progressive (Maher et al., 2020). Overall, these data suggest that long-term oral administration of Pirfenidone does not increase the risk of ADRs, which is consistent with the known safety characteristics of Pirfenidone. Pirfenidone is a small synthetic molecule with high oral bioavailability, which is primarily metabolized via the liver, although the specific mode of metabolism remains unknown. Currently, the pharmacokinetic data obtained are from trials with small sample sizes, and large-scale clinical trial data are still needed (Cho and Kopp, 2010).

## Simtuzumab (GS-6624, INN, SIM)

Simtuzumab is a monoclonal antibody that targets blocking Lysyl Oxidase Like Protein-2 (LOXL2) (Harrison et al., 2018a; Fickert, 2019). LOXL2 is a protease that modifies the ECM by promoting cross-linking of collagen fibers, is believed to play an important role in tumor progression and fibrosis, with the potential to inhibit tumor progression and reverse fibrosis (Figures 2, 3) (Muir et al., 2019; Sanyal et al., 2019).

It has been reported that LOXL2 can promote the occurrence of liver cell fibrosis by catalyzing collagen cross-linking (Meissner et al., 2016; Raghu et al., 2017). Researchers have investigated the safety and efficacy of Simtuzumab in patients with advanced fibrosis caused by NASH (Meissner et al., 2016; Sanyal et al., 2019). In a double-blind study of 219 patients with bridging fibrosis caused by NASH, the patients were randomly assigned (in a 1:1:1 ratio) to a subcutaneous injection of Simtuzumab (75 or 125 mg) or placebo each week for 240 weeks. The experiment was stopped after week 96, and the results showed that liver collagen levels were significantly reduced in all three groups of patients with bridging fibrosis, including those who were given a placebo (Sanyal et al., 2019).

Gilead designed five phase II trials for Simtuzumab to investigate the potential of Simtuzumab in the treatment of pancreatic cancer, colorectal cancer, myeloid fibrosis, IPF, and hepatic fibrosis (Harrison et al., 2018a; Fickert, 2019). To date, two phase II trials of Simtuzumab have failed in pancreatic cancer and colorectal cancer, the remaining three remain to be observed. The latter two indications (pulmonary fibrosis and hepaitc fibrosis) may be the best bet for Simtuzumab. Gilead is currently investigating the efficacy of Simtuzumab for treating IPF (Fickert, 2019; Muir et al., 2019; Sanyal et al., 2019).

A multicenter phase Ⅱ trial evaluated the efficacy and safety of Selonsertib (a selective inhibitor of ASK1) or in combination with Simtuzumab in patients with NASH and stage 2 or 3 hepatic fibrosis (Harrison et al., 2018b). A total of 72 patients were randomized to open treatment for 24 weeks. Patients were treated with 6 mg or 18 mg Selonsertib orally once daily along with or without a weekly injection of 125 mg Simtuzumab, or with Simtuzumab alone. The results showed that hepatic fibrosis improved in 20% of patients treated with Simtuzumab alone after 24 weeks of treatment. The improvement of hepatic fibrosis was related to decreased liver hardness, reduced collagen content and inflammation of liver lobules, as well as an improvement in serum biomarkers of apoptosis and necrosis (Harrison et al., 2018a). There were no significant differences in side effects among the three treatment groups. At present, no pharmacodynamic and pharmacokinetic assays are available to assess whether LOXL2 is indeed effectively inhibited in the human liver, and further studies are needed (Fickert, 2019).

## Sorafenib (BAY43-9006, Nexavar)

Sorafenib is a small molecule compound whose chemical name is 4-[4-({[4-chloro-3-(trifluoromethyl) phenyl] carbamoyl} amino) phenoxy]-Nmethylpyridine-2-carboxamide (Figure 1) (Thabut et al., 2011; Liu et al., 2018), the formula is C_21_H_16_ClF_3_N_4_O_3_, and the molecular weight is 464.82. Sorafenib is an oral multiple kinase inhibitor and a novel multi-molecular target chemotherapy drug. Sorafenib mainly functions by inhibiting tumor cell proliferation, inhibiting angiogenesis, and promoting tumor cell apoptosis (Chen et al., 2014; Deng et al., 2013). Clinically, Sorafenib is mostly used for the treatment of advanced malignant tumors, particularly liver cancer (Lin et al., 2016; Faivre et al., 2020). Sorafenib can prolong the survival of patients with advanced HCC by 3 months on average, but due to congenital or acquired resistance of patients, it is usually not longer than 6 months before Sorafenib resistance occurs (Figures 2, 3) (Faivre et al., 2020).

Hepatic fibrosis, as an important feature of the pathogenesis of chronic liver diseases, has always been one of the important topics in the field of liver disease research and treatment (Ma et al., 2017; Sung et al., 2018). The causes of hepatic fibrosis are complex, TGF-β is the most important known factor promoting hepatic fibrosis (Deng et al., 2013; Ma et al., 2017; Faivre et al., 2020). Hepatic parenchymal cells secrete TGF-β during injury and inflammation, stimulate and activate HSCs, induce epithelial-mesenchymal transition (EMT) of hepatic parenchymal cells, then form activated myofibroblasts and increase the synthesis of ECM such as collagen, thus promoting the occurrence of fibrosis disease (Deng et al., 2013; Lin et al., 2016; Sung et al., 2018). Therefore, therapeutic strategies targeting TGF-β signaling provide the possibility for the eventual prevention and treatment of hepatic fibrosis.

The effective treatment of hepatic fibrosis is an urgent problem to be solved. Studies have shown that combined treatment with Sorafenib and Fluvastatin can reduce collagen deposition and protein expression of α-SMA, down-regulate the content of hyaluronic acid (HA), and the expression of mesenchymal markers in rats with hepatic fibrosis induced by diethylnitrosamine (DEN). Our results suggest that combination therapy can inhibit the progression of hepatic fibrosis by inhibiting the TGF-β1/Smad3 pathway. As such, Sorafenib and fluvastatin may be a potential treatment for hepatic fibrosis. The present study found that Sorafenib can significantly inhibit the TGF-β signal, thereby inhibiting TGF-β-mediated EMT and apoptosis of hepatic parenchymal cells (Thabut et al., 2011; Ma et al., 2017). By establishing a mouse model of hepatic fibrosis induced by CCl_4_, researchers found that feeding Sorafenib to model mice effectively reduced EMT and apoptosis in liver parenchymal cells, and improved and repaired hepatic fibrosis symptoms in mice (Ma et al., 2017; Sung et al., 2018). This work provides a new mechanism for Sorafenib to improve hepatic fibrosis, as well as a theoretical and experimental basis for whether the drug can be finally applied in the clinical treatment of organ fibrosis. Although Sorafenib is a potential therapeutic agent for hepatic fibrosis, it has side effects such as hand-foot syndrome, diarrhea, and hypertension due to oral administration and its non-specific uptake by normal tissues. In addition, the poor water solubility of Sorafenib decreases the efficiency of its absorption by the gastrointestinal tract, leading to poor pharmacokinetics. Future preparation formulation studies of the drug should address these issues (Lin et al., 2016).

## Conclusion and Future Directions

It should be noted that the development of drugs specific to hepatic fibrosis is still in its infancy. Additionally, most drugs are in phase of animal experiments or clinical trials and have various disadvantages, including lack of chronic toxicity, pharmacokinetic studies, and adequate evidence-based medicine. The occurrence and development of hepatic fibrosis is a complex process with many factors and steps. Single drugs often cause obvious adverse reactions due to large doses, single drug targets, and other factors, prices of them are often expensive. Therefore, drug combination has shown advantages of improving efficacy and reducing toxicity in clinical studies, and multi-target anti-hepatic fibrosis drugs are an important direction of future drug R&D.

Commonly used anti-hepatic fibrosis drugs include immunosuppressants, glucocorticoids, and non-specific anti-inflammatory drugs, but these drugs have more adverse reactions and poor efficacy. Moreover, due to individual differences, some patients may have adverse reactions. We found the central event in fibrogenesis appears to be the activation of HSCs, which is a complex process, leading to multiple potential targets for therapeutic interventions. Targeting only one of these targets is difficult to play an anti-hepatic fibrosis effect immediately. With the development of molecular biology, molecular targeted therapy has broad clinical application prospects beyond conventional drug therapy. Additionally, traditional Chinese medicine has unique therapeutic advantages. Therefore, the combined application of different drugs for treating hepatic fibrosis is the direction of future clinical research. Most drugs for hepatic fibrosis are still in the experimental stage. With further research on its formation mechanism and the development of new drugs, hepatic fibrosis may eventually be reversed.
